# Development and validation of an LC‐MS/MS method for quantifying nine antimicrobials in human serum and its application to study the exposure of Chinese pregnant women to antimicrobials

**DOI:** 10.1002/jcla.23658

**Published:** 2020-11-20

**Authors:** Youran Li, Xiaofei Yue, Zhifeng Pan, Ying Liu, Min Shen, Yanhong Zhai, Zheng Cao

**Affiliations:** ^1^ Department of Laboratory Medicine Beijing Obstetrics and Gynecology Hospital Capital Medical University Beijing China; ^2^ Shanghai Trace Source Biotechnology Co., Ltd Shanghai China; ^3^ Reference Laboratory Medical System Biotechnology Co., Ltd Ningbo China

**Keywords:** antimicrobials, Chinese pregnant women, LC‐MS, MS, serum

## Abstract

**Background:**

To study the prevalence of the exposure of pregnant women to antimicrobials, a sensitive and reliable liquid chromatography‐tandem mass spectrometry (LC‐MS/MS) method was developed and validated to determine nine antimicrobials, namely sulfadimidine, sulfapyridine, sulfadiazine, sulfathiazole, ofloxacin, ciprofloxacin, norfloxacin, tetracycline, and lincomycin, in human serum.

**Methods:**

The sample preparation procedure included protein precipitation followed by a cleanup step with solid phase extraction (SPE). Separation was carried out using a CORTECS T3 column (100 × 2.1 mm, 2.7 µm) by gradient elution with a runtime of 8.0 min. Detection was performed on a triple quadruple tandem mass spectrometer with scheduled multiple reaction monitoring (sMRM) in positive ion scan mode.

**Results:**

The calibration curves were linear over the concentration range of 0.5–50 ng/ml, and the limit of quantitation was between 0.01 and 0.2 ng/ml. For each level of quality control samples, the inter‐ and intra‐assay precision values were less than 12.0%, and the accuracy ranged from 86.1% to 109.0%. No significant matrix effect or carryover was observed. The antimicrobials of interest were stable under all investigated conditions. The validated method was applied to analyze clinical samples from pregnant women in China, and 10 out of 500 samples showed the presence of antimicrobial residues. Moreover, compared with the time‐resolved fluoro‐immunoassay (TRFIA) method, the developed method showed greater sensitivity and specificity.

**Conclusion:**

This study provides a simple and rapid LC‐MS/MS method for the simultaneous measurement of nine antimicrobials in serum samples, which could be a useful tool in clinical utilization.

## Graphical abstract

We proposed a simple SPE LC‐MS/MS method for the analysis of nine antimicrobials in serum. The elaborated method was applied to investigate the exposure of antimicrobials in Chinese pregnant women, and the results have been compared with those obtained from TRFIA method.

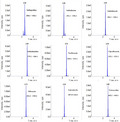

## 1 INTRODUCTION

As an effective and powerful regimen, antimicrobials have been widely used in humans, animals, and plants for preventing or treating infection, as well as for promoting growth.^1^ Consequently, this extensive application has been accompanied by a large amount of antimicrobials disposed into the environment, which may be from pharmaceutical processes or excreted as parent compounds or metabolites due to poor gut absorption or incomplete metabolism.^2^, ^3^ These compounds are eventually directly or indirectly introduced into the human body by contaminated soil, food, and water, thus posing a potential risk to public health.^4^, ^5^

It is evident that the presence of antimicrobials at low levels is associated with several adverse health issues, such toxic effects, allergic reactions, and the development of bacterial resistance.^6^, ^7^ The impacts of antimicrobial exposure on pregnant women would be exacerbated, as antimicrobials could be transmitted from mother to infant via the blood‐placenta barrier or through breastfeeding.^8^ Antimicrobials can also have an impact on the human intestinal flora. The gut bacterial community has many critical functions, such as aiding in the development of the immune system and the metabolic system^9^, ^10^ and in the synthesis of vitamin K. In the early stage after birth, the development of a stable microbial community is essential.^11^ Antimicrobial exposure in infancy would disrupt the microbiome composition in the gut, which would have long‐lasting effects on the host physiology.^12^ Therefore, it is necessary to develop an analytical method capable of detecting multiclass antimicrobials to better investigate antimicrobial exposure levels in pregnant women.

To date, several analytical methods have been used to detect antimicrobials, including microbiological inhibition assays, immunoassays, biosensors, and liquid chromatography‐tandem mass spectrometry (LC‐MS/MS),^13^, ^14^ among which LC‐MS/MS has become a preferred technique due to its high selectivity and sensitivity. However, only a few LC‐MS/MS methods available for the comprehensive analysis of a panel of antimicrobials in human samples have been reported.^15^, ^16^ Most methods focus on the analysis of one antimicrobial or a single class of antimicrobials. Furthermore, those methods were used for therapeutic drug monitoring (TDM) or for pharmacokinetic studies. However, the exposure level of antimicrobials is much lower than the drug concentration in blood, and the species count is much higher because medical and veterinary antimicrobials are all included.

To quickly monitor trace antimicrobial exposure levels in pregnant women, we developed a rapid and sensitive multiclass method capable of detecting nine antimicrobials (four sulfonamides, three quinolones, one tetracycline, and one lincosamide) in human serum in one analytical run. The selected antimicrobials are the most commonly detected in environmental and food safety tests reported in the literature,^2^, ^17^, ^18^ not only those that are commonly used in clinical therapy. The method was fully verified and then successfully applied to the analysis of antimicrobials in serum samples from pregnant women. The reliability of the proposed method was further verified by comparison with the traditional time‐resolved fluoro‐immunoassay (TRFIA) method.

## 2 MATERIALS AND METHODS

### 2.1 Chemicals and materials

The antimicrobial standards of sulfadimidine, sulfapyridine, sulfadiazine, sulfathiazole, ofloxacin, ciprofloxacin, norfloxacin, tetracycline, and lincomycin were purchased from the National Institutes for Food and Drug Control. The internal standards (ISs) of norfloxacin‐d_8_, sulfadimidine‐d_4_, tetracycline‐d_6_, and lincomycin‐d_3_ were purchased from TRC. LC‐MS‐grade acetonitrile (ACN), methanol (MeOH), and formic acid (FA) were purchased from Fisher Scientific. Analytical reagent grade phosphoric acid was obtained from Sigma‐Aldrich. Oasis HLB μEluting Plates used for solid phase extraction (SPE) were obtained from Waters Corporation. The TRFIA kits were provided by Shanghai Tracing Biotech Company Limited.

### 2.2 Preparation of calibrators, quality control samples, and internal standards

Stock solutions of the antimicrobials were individually prepared at a concentration of 1 mg/ml either in MeOH or water. An appropriate amount of each stock solution was spiked into 60% (v/v) ACN and diluted with 10% (v/v) ACN to obtain the mixed working solution series. A seven‐point calibrator was prepared by spiking mixed working solutions into blank serum, and the final concentration of the calibrator was 0.5, 1, 2.5, 5, 10, 25, and 50 ng/ml for all the analytes. The quality control (QC) samples were prepared at low (1 ng/ml), medium (20 ng/ml), and high (40 ng/ml) levels for each analyte.

The working internal standard solution was prepared as a mixture, and the concentrations used were 1 µg/ml for norfloxacin‐d_8_, 0.2 µg/ml for sulfadimidine‐d_4_, 0.4 µg/ml for tetracycline‐d_6_, and 0.2 µg/ml for lincomycin‐d_3_.

### 2.3 Sample preparation

For each 200 µl serum sample, 190 µl of phosphoric acid and 10 µl of the IS mixture solution were added for protein precipitation. After vortexing the mixtures, 300 µl of supernatant was collected and loaded into an SPE cartridge. The SPE cartridge was then washed twice with 150 µl water before elution with 150 µl 60% (v/v) ACN. The eluate was collected in a clean test tube, and 2 µl of the eluate of each sample was injected into the LC‐MS/MS system for analysis.

### 2.4 Instrumental analysis

The sample analyses were performed using a Shimadzu LC‐20AD HPLC system coupled with an ABSCIEX 5500 triple quadrupole mass spectrometer equipped with an electrospray ionization (ESI) source. Chromatographic separation was performed with a Waters CORTECS T3 column (100 × 2.1 mm, 2.7 µm). The column temperature was kept at 35°C. The mobile phase was composed of water containing 0.1% formic acid (A) and ACN containing 0.1% formic acid (B). The gradient program was set as follows: 0 min 5% B, 5 min 60% B, 5.5 min 90% B, 5.5 ~ 6.5 min 90% B, and 6.6 ~ 8 min 5% B. The flow rate was set at 0.3 ml/min, and the injection volume was 2 µl.

The MS was operated in positive ion mode with sMRM. The optimized MS parameters for each antimicrobial are summarized in Table 1. The parameters of the source conditions were as follows: ion spray (IS) voltage: 4500 V; curtain gas: 20 psi; nebulizer gas (GS1): 50 psi; auxiliary gas (GS2): 50 psi; and source temperature: 500°C. Data acquisition was performed by Analyst 1.6.3 software. The peak integration and data analysis were processed by MultiQuant software (version 3.0.2).

**TABLE 1:** Acquisition parameters of the analytes

Analyte	Precursor/product ion pairs (*m/z*)	MS/MS parameter (v)	
		DP	CE
Sulfapyridine	250.2/156.1^*^	80	23
	250.2/184.1		
Sulfadiazine	251.2/156.1^*^	70	22
	251.2/92.2		
Sulfathiazole	256.1/156.1^*^	60	21
	256.1/108.1		
Sulfadimidine	279.2/186.1^*^	80	24
	279.2/156.1		
Norfloxacin	320.4/231.1^*^	100	50
	320.4/282.0		
Ciprofloxacin	332.1/231.1^*^	100	45
	332.1/245.0		
Ofloxacin	362.4/318.4^*^	100	28
	362.4/261.3		
Lincomycin	407.0/126.0^*^	100	38
	407.0/359.0		
Tetracycline	445.2/410.1^*^	100	23
	445.2/268.9		

Abbreviations: CE, collision energy; DP, declustering potential.

^*^ indicates ion pairs used for quantification.

### 2.5 Method validation

The method validation was performed in accordance with the recommendations published in the Clinical and Laboratory Standards Institute (CLSI) C62‐A: Liquid Chromatography‐Mass Spectrometry Methods.^19^ The method validation included the specificity, linearity, sensitivity, accuracy, precision, matrix effects, recovery, carryover, and stability.

The specificity was investigated by evaluating blank human serum samples from six different subjects and checking for interfering peaks on the chromatogram of the analytes and ISs.

A least‐squares regression model was used to check the linearity of the method. The linearity was evaluated by building the calibration curves in human serum at seven concentration levels. Calibration curves were constructed by plotting the theoretical standard concentration vs. the peak area ratio of the standard to the IS. The limit of quantification (LOQ) was determined as the concentration at which the signal‐to‐noise (S/N) ratio was >10.

Quality control samples prepared at three different concentrations were used to measure the intra‐ and interday precision and accuracy. The intraday precision was determined by running each QC sample in six replicates on 1 day, and the interday precision was determined by analyzing six replicates of each QC sample on three successive days. The inter‐ and intraday precision values were expressed as the coefficient of variation (CV). Accuracy was calculated as the measured concentration when compared to the nominal concentration of each QC and expressed as a percentage.

The recovery was estimated by comparing the peak areas of pre‐extraction spiked serum with those obtained from postextraction samples at the three QC concentrations. The matrix effect was estimated by comparing the peak response of the postextraction spiked serum with the neat solvent standards at the three QC concentrations.

The carryover was assessed by running 4 low‐level QC samples immediately after running 3 high‐level QC samples. The following order is H_1_, H_2_, H_3_, L_1_, L_2_, L_3_, and L_4_. Carryover was calculated as [L_1_‐(L_3_ + L_4_)/2]/[(H_2_ + H_3_)/2‐(L_3_ + L_4_/2)]*100, and the acceptance criteria must be <1%.

The stability studies were performed using spiked serum at low and high QC concentrations (*n* = 6) under the following conditions: in the autosampler at 4°C for 24 h, or after undergoing three freeze‐thaw cycles at −20°C. The stability was evaluated by calculating the difference between the examined samples and the freshly prepared samples.

### 2.6 Method application and comparison

In total, five hundred serum samples from pregnant women (in the second or third trimester, 18–38 years old) who were volunteers from Beijing Obstetrics and Gynecology Hospital were used to compare the performance of the developed LC‐MS/MS method and TRFIA kits. The collected samples were kept at −80°C until analysis. Research related to human use was carried out in accordance with the tenets of the Helsinki Declaration. Ethical approval from the Beijing Obstetrics and Gynecology Hospital Research Ethics Committee and informed consent from patients were obtained.

## 3 RESULTS AND DISCUSSION

### 3.1 Chromatography and mass spectrometry

Regarding the mass spectrometry conditions, the selection of the precursor and product ion was first carried out by direct infusion of standard solutions of each individual target antimicrobial compound. All the compounds of interest were sensitive in positive ion mode of the electrospray ionization source. The protonated ion [M + H]^+^ was predominant in the MS spectrum and was selected as the precursor ion. From the MS/MS spectrum, the two most intense transitions of each compound were selected for operation in sMRM mode: one transition was used for quantification (marked with “*”), and the other was used for qualitative confirmation. After MRM transitions were identified, other MS parameters, such as the source and compound‐dependent parameters, were optimized using the flow injection analysis method.

**FIGURE 1:**
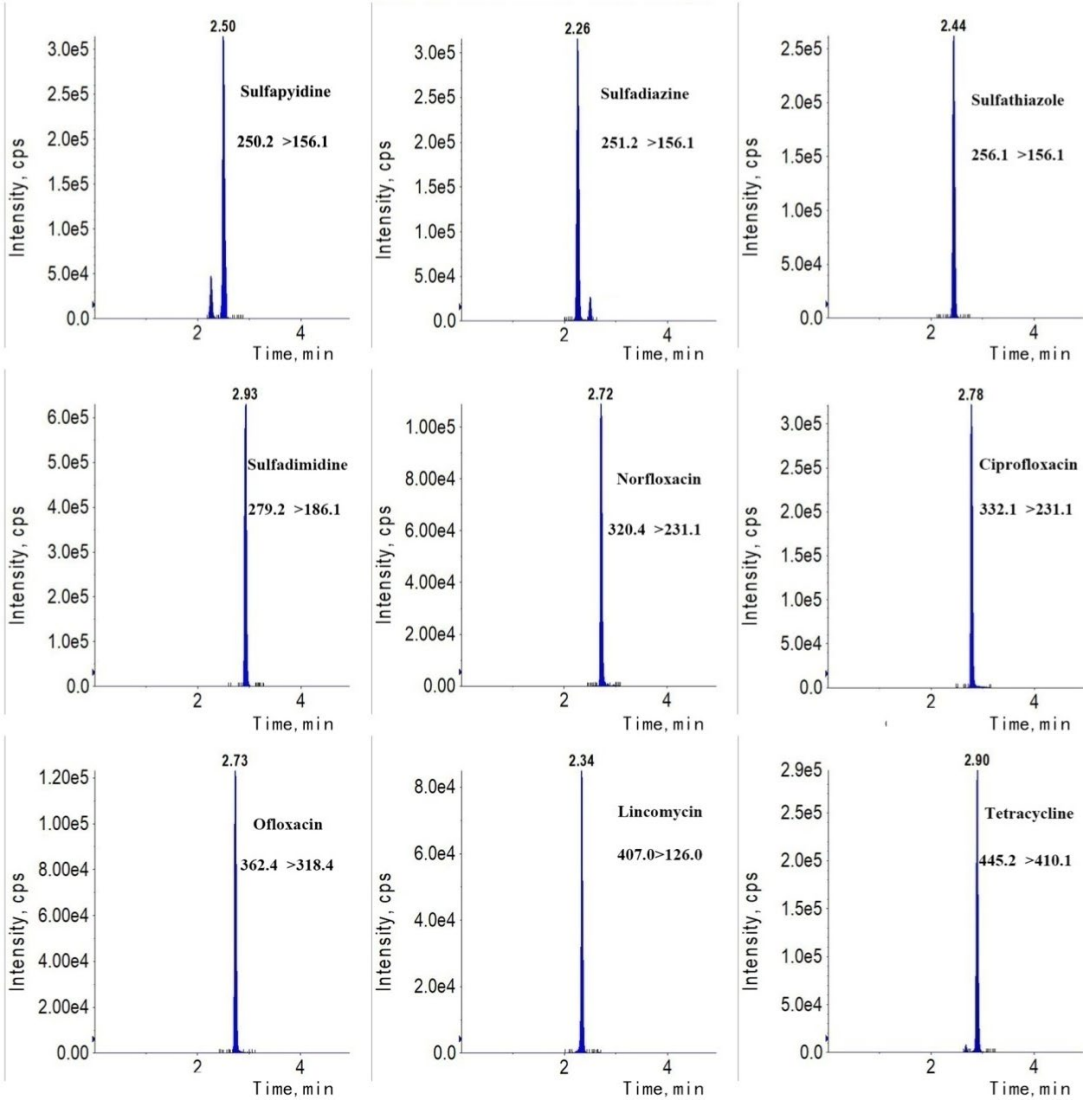
Typical MRM chromatograms of all analytes at a spiking concentration of 5 ng/ml

The analytical system was optimized to achieve the optimum separation, suitable analytical time, and symmetric peak shapes for LC‐MS/MS analysis. In the methodology development phase, an Agela Venusil MP C18 column (50 × 3.0 mm, 3.0 µm), a Waters CORTECS T3 column (100 × 2.1 mm, 2.7 µm), and a Phenomenex Kinetex XB‐C18 column (50 × 2.1 mm, 5.0 µm) were tested. Among the columns evaluated, the CORTECS T3 column could provide excellent peak shapes for all analytes. Therefore, the CORTECS T3 column was used in our further assay. Regarding the selection of organic solvent, MeOH and ACN with different proportions of formic acid (0.02%, 0.1%, 0.2%) were tested. In this study, ACN could result in narrower peak shapes and lower column pressure than MEOH. As a common additive reagent, formic acid is often included in the mobile phase to provide protons and yield improved ionization efficiency.^20^ The results revealed that 0.1% formic acid will increase the MS response and improve the peak shape, while a formic acid content that is too high has no obvious effect. Consequently, ACN containing 0.1% formic acid was used as the optimal organic phase. After optimizing the gradient elution program, nine target antimicrobials were finally analyzed in an 8‐min chromatographic run with suitable separation and good symmetric peaks. A representative chromatogram showing typical peak shapes for each antimicrobial is presented in Figure 1.

In hospital laboratories, quick and easy sample preparation, short run times, and simultaneous measurement of multiple analytes are important for constructing a method to meet the needs of routine applications. In this work, we established a fast and sensitive LC‐MS/MS method for the simultaneous quantification of nine different antimicrobials that are commonly used in humans and agriculture. The ability to determine several antibiotics from different classes in one method is advantageous because it enables different antimicrobials in the samples to be analyzed together at the same time, which substantially increases the practicality of using the method in the future. In addition, the combination of high‐throughput sample preparation (96‐well SPE) and short chromatographic time can minimize sample turnover time, making the method suitable for rapid screening of samples. Furthermore, the use of tandem mass spectrometry and stable isotope‐label IS allows better selectivity, sensitivity, and reproducibility, thus reducing the risk of interference from a biological matrix and analytical variations.

To evaluate the performance of the proposed method, a variety of parameters, including the specificity, linearity of the calibration curve, accuracy and precision, recovery, matrix effects, and stability, were critically evaluated. No chromatographic interference was observed at the retention time of the target compounds, indicating good specificity.

Linearity was investigated by preparing matrix‐matched calibration curves. The calibration curves were fitted using linear regression with a weighting factor of 1/x^2^. Over the calibration range selected (0.5–50 ng/ml), the method was significantly linear according to the coefficient values (r^2^ > 0.99) for all the analytes. The LOQs of all the analytes were lower than 0.2 ng/ml. The results obtained by using this method with human serum were linear and sensitive for each analyte. The regression parameters and LOQs are summarized in Table 2.

**TABLE 2:** Calibration range, LOQ, matrix effect and recovery of the analytes

Analyte	Range (ng/ml)	R^2^	LOQ (ng/ml)	Matrix effect (%)			Recovery (%)		
				1 ng/ml	10 ng/ml	40 ng/ml	1 ng/ml	10 ng/ml	40 ng/ml
Sulfapyridine	0.5 ~ 50	0.995	0.02	89.47	102.13	104.76	85.72	78.21	94.73
Sulfadiazine	0.5 ~ 50	0.998	0.05	95.37	89.94	85.98	68.52	75.76	90.47
Sulfathiazole	0.5 ~ 50	0.995	0.10	89.61	93.19	88.33	60.05	61.70	82.67
Sulfadimidine	0.5 ~ 50	0.997	0.02	95.02	87.74	79.77	74.70	78.14	92.97
Norfloxacin	0.5 ~ 50	0.997	0.05	95.15	91.97	94.54	93.92	101.81	101.57
Ciprofloxacin	0.5 ~ 50	0.993	0.01	80.11	81.69	80.03	79.61	82.51	88.17
Ofloxacin	0.5 ~ 50	0.991	0.10	86.43	90.54	87.27	47.94	49.86	49.95
Lincomycin	0.5 ~ 50	0.996	0.20	95.51	101.50	103.25	16.43	15.70	19.25
Tetracycline	0.5 ~ 50	0.999	0.05	85.80	84.46	81.97	89.14	92.88	111.42

Intraday and interday precision and accuracy were assessed at the three QC levels. Data on accuracy and precision for each analyte in serum are presented in Table 3. The accuracy for all the compounds was between 86.1% and 109.0%. The intra‐and interday precisions for all the analytes ranged from 3.70% to 11.5% and 5.18% to 10.3%, within the 15% limit requested. These results indicated that this method gave satisfactory precision and accuracy for all the antimicrobials obtained from human serum.

**TABLE 3:** Accuracy and precision of the analytes

Analyte	Interday precision (CV%)			Intraday precision (CV%)			Accuracy (%)		
	1 ng/ml	10 ng/ml	40 ng/ml	1 ng/ml	10 ng/ml	40 ng/ml	1 ng/ml	10 ng/ml	40 ng/ml
Sulfapyridine	3.70	9.12	8.01	7.51	9.37	8.27	94.67	102.23	94.56
Sulfadiazine	8.78	8.74	8.81	6.81	6.71	7.17	109.00	96.88	95.55
Sulfathiazole	9.86	8.09	6.64	8.89	9.18	10.32	97.67	90.70	87.95
Sulfadimidine	8.17	4.15	6.47	7.89	6.25	6.07	101.50	91.37	91.85
Norfloxacin	6.91	5.47	4.92	9.33	6.89	8.57	97.08	101.28	90.62
Ciprofloxacin	6.98	5.22	3.96	7.18	6.26	5.18	96.67	105.05	97.09
Ofloxacin	11.50	6.77	2.06	10.24	6.43	8.76	102.67	95.80	86.13
Lincomycin	4.30	5.25	8.03	6.81	7.51	7.51	99.50	99.77	93.57
Tetracycline	5.08	7.01	8.42	6.03	6.50	5.75	99.17	99.25	100.22

The matrix effect is caused by coeluting components from patient sera, resulting in ion suppression or enhancement. The matrix effect was evaluated with three levels of QCs. As shown in Table 2, only minor differences were observed between the pure standards and the postextraction spiked samples, indicating little influence of the background signal of serum on analytes. The recovery for each analyte was greater than 60.0% except for ofloxacin and lincomycin (Table 2). The carryover of all the antibiotics was within the minimum level (< 1%) (data not shown).

The inaccuracy for freeze‐thaw samples ranged from −14.8% to 10.8%. The inaccuracy of autosampler stability ranged from −12.8% to 7.82%, showing good stability under postextracted sample storage conditions for 24 h at 4°C (Table S1).

The performance characteristics of the method are summarized above. More than nine antimicrobials were included in the preliminary experiments, but some of them were not successfully validated for several reasons, such as instability or lack of a suitable IS. There is much work that needs to be performed in future work with the aim of analyzing more antimicrobials.

### 3.2 Application and comparison

The optimized and validated method was applied to screen antimicrobial residues in serum samples of pregnant women recruited from Beijing Obstetrics and Gynecology Hospital. Among the 500 samples, 10 samples were positive for the target antimicrobials, and the overall detection frequency was 2.0% (Table 4). The detection frequency of the nine antimicrobials varied from 0.2% to 1.0%. Specifically, ofloxacin and sulfadiazine were the most frequently detected antimicrobials in the serum of pregnant women, accounting for 52.1% and 30.4%, respectively. The highest value was above 50 ng/ml. The lowest concentration in serum was found for lincomycin (0.67 ng/ml). At least two antimicrobials were detected simultaneously in some samples, for example, sample 1 sulfapyridine + sulfadiazine +lincomycin.

**TABLE 4:** Occurrence of antimicrobial residues in the serum samples of pregnant women analyzed using the TRFIA and LC‐MS/MS methods

Sample ID	TRFIA	LC‐MS/MS
1	Sulfonamides	Sulfapyridine (2.57 ng/ml)
		Sulfadiazine (>50 ng/ml)
		Lincomycin (0.67 ng/ml)
2	Sulfonamides	Sulfadiazine (>50 ng/ml)
		Sulfathiazole (0.76 ng/ml)
		Sulfadimidine (1.65 ng/ml)
		Ofloxacin (12.57 ng/ml)
		Lincomycin (3.91 ng/ml)
3	Sulfonamides	Sulfapyridine (>50 ng/ml)
		Ofloxacin (>50 ng/ml)
4	Sulfonamides	Sulfadiazine (1.34 ng/ml)
		Sulfadimidine (37.27 ng/ml)
		Ofloxacin (7.19 ng/ml)
5	Tetracycline	Sulfapyridine (0.65 ng/ml)
		Sulfadiazine (4.39 ng/ml)
		Tetracycline (>50 ng/ml)
6	Quinolones	Ofloxacin (>50 ng/ml)
7	Quinolones	Ciprofloxacin (>50 ng/ml)
8	Tetracycline	Sulfapyridine (0.91 ng/ml)
		Ofloxacin (4.05 ng/ml)
		Tetracycline (1.8 ng/ml)
9	n.d.	Sulfadiazine (2.38 ng/ml)
10	Quinolones	Norfloxacin (>50 ng/ml)

Abbreviation: n.d, not detected.

The women enrolled in this study were not under clinical treatment with antimicrobials, and the antimicrobials detected by the LC‐MS/MS method might be from exposure to contaminated foods or the environment. There are limited studies to comprehensively evaluate the antimicrobial exposure levels in pregnant women. Zeng et al^21^ reported that sulfonamides and quinolones were found to be the most abundant in the urine of pregnant women, which was in accordance with the results obtained in our study. Cumulative exposure to antibiotics during pregnancy may adversely affect the health of mothers and offspring.

The present method showed higher precision and accuracy values compared with TRFIA for determining antimicrobials in human serum. The developed LC‐MS/MS method detected more antimicrobials than the TRFIA method. For instance, lincomycin was undetectable in samples 1 and 2, while sulfonamides were not detected in samples 5, 8, and 9 with the TRFIA method. In addition, LC‐MS/MS has the ability to analyze a panel of drugs and confirm specific antimicrobial residues. The principle of TRFIA is the specific immunological reaction between antibody and antigen, but this method suffers from poor specificity because of interferences.^22^ The LC‐MS/MS method utilizes multiple mass‐resolving devices to eliminate the chemical background, providing improved quantification capabilities of multiple known compounds as well as the ability to qualitatively analyze unknowns in biological samples.^23^ Therefore, the use of tandem mass spectrometry allows for better selectivity and sensitivity than traditional TRFIA. The comparison results further exhibited the advantage of the developed method for use in clinical practice.

## 4 CONCLUSIONS

In this work, a powerful method for the simultaneous analysis of nine antimicrobials in human serum that are most frequently found in environmental or agricultural products was developed and optimized. The proposed method is rapidly performed and is reliable, and it allows the determination of multiclass antimicrobials belonging to the sulfonamide, quinolone, lincosamide, and tetracycline families in a single run within a short time of 8 min. The assay was successfully validated in terms of sensitivity, linearity, accuracy, precision, recovery, carryover, and stability. Serum samples from pregnant women were analyzed to evaluate the applicability of this method. At least one antibiotic was detected in some samples, and the species accounting for the largest proportions were similar to those found in a previous study. Comparisons of the performance in screening antimicrobials with the TRFIA technique strongly support the sensitivity and selectivity of our presented method. These results indicated that the present method is practical and effective and can be used in clinical laboratories to assess antimicrobial residues to protect pregnant women's health. However, we note the limitations of this work, such as the slightly tedious sample preparation and the insufficient number of antimicrobials. Future studies should include approaches to simplify the sample treatment procedure and incorporate as many antimicrobials as possible.

## Supporting information

Tab S1

## Data Availability

The data during and/or analyzed during the current study are available from the corresponding author on reasonable request.

## References

[1] Zhu S , Chen H , Li J . Sources, distribution and potential risks of pharmaceuticals and personal care products in Qingshan Lake basin, Eastern China. Ecotoxicol Environ Saf. 2013;96:154‐159. 23871206 10.1016/j.ecoenv.2013.06.033

[2] Zhang QQ , Ying GG , Pan CG , Liu YS , Zhao JL . Comprehensive evaluation of antibiotics emission and fate in the river basins of China: source analysis, multimedia modeling, and linkage to bacterial resistance. Environ Sci Technol. 2015;49:6772‐6782. 25961663 10.1021/acs.est.5b00729

[3] Liu X , Lu S , Guo W , Xi B , Wang W . Antibiotics in the aquatic environments: a review of lakes, China. Sci Total Environ. 2018;627:1195‐1208. 30857084 10.1016/j.scitotenv.2018.01.271

[4] Fabio K , Shlomo EB . The occurrence of veterinary pharmaceuticals in the environment: a Review. Curr Anal Chem. 2016;12:169‐182. 28579931 10.2174/1573411012666151009193108 PMC5425647

[5] Larsson DG . Antibiotics in the environment. Ups J Med Sci. 2014;119:108‐112. 24646081 10.3109/03009734.2014.896438 PMC4034546

[6] Liu X , Steele JC , Meng XZ . Usage, residue, and human health risk of antibiotics in Chinese aquaculture: a review. Environ Pollut. 2017;223:161‐169. 28131482 10.1016/j.envpol.2017.01.003

[7] Kim C , Ryu HD , Chung EG , Kim Y , Lee JK . A review of analytical procedures for the simultaneous determination of medically important veterinary antibiotics in environmental water: sample preparation, liquid chromatography, and mass spectrometry. J Environ Manage. 2018;217:629‐645. 29649735 10.1016/j.jenvman.2018.04.006

[8] Lemas DJ , Yee S , Cacho N , et al. Exploring the contribution of maternal antibiotics and breastfeeding to development of the infant microbiome and pediatric obesity. Semin Fetal Neonatal Med. 2016;21:406‐409. 27424917 10.1016/j.siny.2016.04.013

[9] Sampson TR , Mazmanian SK . Control of brain development, function, and behavior by the microbiome. Cell Host Microbe. 2015;17:565‐576. 25974299 10.1016/j.chom.2015.04.011 PMC4442490

[10] Rautava S , Luoto R , Salminen S , Isolauri E . Microbial contact during pregnancy, intestinal colonization and human disease. Nat Rev Gastroenterol Hepatol. 2012;9:565‐576. 22890113 10.1038/nrgastro.2012.144

[11] Trasande L , Blustein J , Liu M , Corwin E , Cox LM , Blaser MJ . Infant antibiotic exposures and early‐life body mass. Int J Obes (Lond). 2013;37(1):16‐23. 22907693 10.1038/ijo.2012.132 PMC3798029

[12] Cox LM , Blaser MJ . Antibiotics in early life and obesity. Nat Rev Endocrinol. 2015;11:182‐190. 25488483 10.1038/nrendo.2014.210 PMC4487629

[13] Cháfer‐Pericás C , Maquieira Á , Puchades R . Fast screening methods to detect antibiotic residues in food samples. TrAC, Trends Anal Chem. 2010;29:1038‐1049.

[14] Delatour T , Racault L , Bessaire T , Desmarchelier A . Screening of veterinary drug residues in food by LC‐MS/MS. Background and challenges. Food Addit Contam Part A. 2018;35:632‐645. 10.1080/19440049.2018.1426890 29324075

[15] Barco S , Mesini A , Barbagallo L , et al. A liquid chromatography‐tandem mass spectrometry platform for the routine therapeutic drug monitoring of 14 antibiotics: application to critically ill pediatric patients. J Pharm Biomed Anal. 2020;186:113273. 32251979 10.1016/j.jpba.2020.113273

[16] Abdulla A , Bahmany S , Wijma RA , et al. Simultaneous determination of nine β‐lactam antibiotics in human plasma by an ultrafast hydrophilic‐interaction chromatography–tandem mass spectrometry. J Chromatogr B. 2017;1060:138‐143. 10.1016/j.jchromb.2017.06.014 28618388

[17] He ZX , Cheng XR , Kyzas GZ , Fu J . Pharmaceuticals pollution of aquaculture and its management in China. J Mol Liq. 2016;223:781‐789.

[18] Baynes RE , Dedonder K , Kissell L , et al. Health concerns and management of select veterinary drug residues. Food Chem Toxicol. 2016;88:112‐122. 26751035 10.1016/j.fct.2015.12.020

[19] CLSI . Liquid chromatogtaphy‐mass spectrometry methods; approved guideline, CLSI document C62‐A. Wayne, PA: Clinical and Laboratory Standards Institute; 2014.

[20] Wu JB , Qian YS , Zhang CL , et al. Application of graphene‐based solid‐phase extraction coupled with ultra high‐performance liquid chromatography‐tandem mass spectrometry for determination of macrolides in fish tissues. Food Anal Methods. 2013;6:1448‐1457.

[21] Zeng X , Zhang L , Chen Q , et al. Maternal antibiotic concentrations in pregnant women in Shanghai and their determinants: a biomonitoring‐based prospective study. Environ Int. 2020;138:105638. 32179319 10.1016/j.envint.2020.105638

[22] Gaudin V . Advances in biosensor development for the screening of antibiotic residues in food products of animal origin ‐ A comprehensive review. Biosens Bioelectron. 2017;90:363‐377. 27940240 10.1016/j.bios.2016.12.005

[23] Valese AC , Molognoni L , de Souza NC , et al. Development, validation and different approaches for the measurement uncertainty of a multi‐class veterinary drugs residues LC‐MS method for feeds. J Chromatogr B. 2017;1053:48‐59. 10.1016/j.jchromb.2017.03.026 28411464

