# Supplementary material for: Development and validation of an LC‐MS/MS method for quantifying nine antimicrobials in human serum and its application to study the exposure of Chinese pregnant women to antimicrobials
Source: J Clin Lab Anal. 2020 Nov 20;35(3):e23658. doi: 10.1002/jcla.23658 (PMC7958000; doi:10.1002/jcla.23658)
Supplement: Supplementary file 1 — Tab S1 [file JCLA-35-e23658-s001.docx]

**Supporting information**

**Development and validation of an LC-MS/MS method for quantifying nine antimicrobials in human serum and its application to study the exposure of Chinese pregnant women to antimicrobials**

Youran Li^1#^, XiaofeiYue^1#^, Zhifeng Pan^2^, Ying Liu^1^, Min Shen^3^, Yanhong Zhai^1^, Zheng Cao^1*^

1Department of Laboratory Medicine, Beijing Obstetrics and Gynecology Hospital, Capital Medical University, Beijing, China

2 Shanghai Trace Source Biotechnology Co., Ltd, Shanghai, China

3Reference Laboratory, Medical System Biotechnology Co., Ltd, Ningbo, Zhejiang, China

^#^Contributed equally to this work.

* Corresponding author. Address: Department of Laboratory Medicine, Beijing Obstetrics and Gynecology Hospital, Capital Medical University, 251 Yaojiayuan Road, Beijing, China 100026.

Tel.: 86-10-52276406; Email: [zhengcao2011@hotmail.com](mailto:zhengcao2011@hotmail.com)

**Table S-1. Stability of the analytes**

**TABLES**

| **TABLE S-1** Stability of the analytes | | | | | | | | | | | |
| --- | --- | --- | --- | --- | --- | --- | --- | --- | --- | --- | --- |
| **Ananyte** | **4 ℃ for 24h (%)** | | | |  | | | **3 Freeze-thaw cycles** | | | |
|  | **1 ng/mL** | **10 ng/mL** | **40 ng/mL** | |  | | **1 ng/mL** | | **10 ng/mL** | **40 ng/mL** | |
| Sulfapyridine | 6.20 | 7.82 | 7.50 |  | | 8.00 | | | 3.70 | | 10.77 |
| Sulfadiazine | -11.40 | -8.68 | -6.72 |  | | 0.80 | | | -0.86 | | 7.24 |
| Sulfathiazole | -0.20 | -6.62 | -8.35 |  | | 0.20 | | | -9.06 | | -0.64 |
| Sulfadimidine | -4.40 | -8.40 | -6.36 |  | | 1.20 | | | -1.22 | | 5.67 |
| Norfloxacin | -0.80 | -5.14 | -7.56 |  | | -11.80 | | | -14.84 | | -9.96 |
| Ciprofloxacin | 5.20 | -8.22 | -10.36 |  | | -11.20 | | | -12.32 | | -9.15 |
| Ofloxacin | -12.00 | -11.64 | -12.76 |  | | -1.60 | | | -8.38 | | -3.92 |
| Lincomycin | -5.00 | -2.86 | -5.12 |  | | 3.80 | | | -2.00 | | -8.34 |
| Tetracycline | -1.40 | -0.66 | -3.86 |  | | 7.80 | | | -3.54 | | 0.91 |
